# Specialized inpatient treatment of adult anorexia nervosa: effectiveness and clinical significance of changes

**DOI:** 10.1186/s12888-014-0258-z

**Published:** 2014-09-06

**Authors:** Sandra Schlegl, Norbert Quadflieg, Bernd Löwe, Ulrich Cuntz, Ulrich Voderholzer

**Affiliations:** Department of Psychiatry and Psychotherapy, University Hospital of Munich (LMU), Munich, Germany; Department of Psychosomatic Medicine and Psychotherapy, University Medical Center Hamburg-Eppendorf, Hamburg, Germany; Schoen Klinik Hamburg Eilbek, Hamburg, Germany; Schoen Klinik Roseneck, Prien, Germany; Paracelsus Medical University, Salzburg, Austria; Department of Psychiatry and Psychotherapy, University Hospital of Freiburg, Freiburg, Germany

**Keywords:** Anorexia nervosa, Cognitive behavioural therapy, Inpatient, Effectiveness, Clinical significance

## Abstract

**Background:**

Previous studies have predominantly evaluated the effectiveness of inpatient treatment for anorexia nervosa at the group level. The aim of this study was to evaluate treatment outcomes at an individual level based on the clinical significance of improvement. Patients’ treatment outcomes were classified into four groups: deteriorated, unchanged, reliably improved and clinically significantly improved. Furthermore, the study set out to explore predictors of clinically significant changes in eating disorder psychopathology.

**Methods:**

A total of 435 inpatients were assessed at admission and at discharge on the following measures: body-mass-index, eating disorder symptoms, general psychopathology, depression and motivation for change.

**Results:**

20.0-32.0% of patients showed reliable changes and 34.1-55.3% showed clinically significant changes in the various outcome measures. Between 23.0% and 34.5% remained unchanged and between 1.7% and 3.0% deteriorated. Motivation for change and depressive symptoms were identified as positive predictors of clinically significant changes in eating disorder psychopathology, whereas body dissatisfaction, impulse regulation, social insecurity and education were negative predictors.

**Conclusions:**

Despite high rates of reliable and clinically significant changes following intensive inpatient treatment, about one third of anorexia nervosa patients showed no significant response to treatment. Future studies should focus on the identification of non-responders as well as on the development of treatment strategies for these patients.

**Electronic supplementary material:**

The online version of this article (doi:10.1186/s12888-014-0258-z) contains supplementary material, which is available to authorized users.

## Background

Anorexia nervosa is characterized by a pronounced self-induced weight loss or insufficient weight gain during the period of growth and is associated with an irrational fear of gaining weight as well as a conspicuous distortion of body image. This disorder is relatively common among young women. While the overall incidence rate has remained stable over the past decades, there has been an increased incidence in 15-19 year old girls, a group at elevated risk [1]. The disorder is often characterized by a chronic course and elevated mortality rates [2,3] and thus is one of the diseases with the worst prognosis in psychiatry and psychosomatic medicine. Recent state of the art studies suggest that treatment of anorexia nervosa in various settings (outpatient, inpatient, day clinic) is effective [4–6]. In a large randomised controlled trial investigating the efficacy of outpatient treatment of anorexia nervosa significant weight gain as well as reduction in general and eating disorder-specific psychopathology was found [4]. For severe cases of anorexia nervosa with high medical and/or psychosocial risk and a lack of response to less intensive treatments, existing practice guidelines advise inpatient treatment [7–9].

So far, statistical group comparisons have predominantly served to evaluate the effectiveness of inpatient treatment for anorexia nervosa [10–13]. This approach guarantees that the differences found are not based on chance. Yet, it does not provide any information about the variability of response rates within a sample. In addition, statistical significance does not enable conclusions to be drawn about whether or not a change in symptoms is clinically relevant [14]. Effect sizes (ES) are better suited to evaluate therapeutic effects because they provide information about the size of a change and are independent of sample size. However, this measure is also relatively independent of the clinical relevance of results [14]. Authors such as Long et al. [15], Rø et al. [16], Tagay et al. [17], Zeeck et al. [18] and Goddard et al. [19] reported small to high effect sizes of inpatient treatment for anorexia nervosa depending on the outcome measure.

To overcome the shortcomings of measuring treatment outcome in terms of statistical significance or effect sizes, Jacobson and colleagues [14,20] developed a concept that considers the clinical relevance of treatment change on an individual basis.

To our knowledge, there is only one study that reports clinically significant changes in inpatients with eating disorders based on the criteria of Jacobson and Truax [14]. Calugi and colleagues [21] found that after treatment 36.4% of patients with longstanding eating disorder (≥ 10 years) showed clinically significant changes in the Global score of the Eating Disorder Examination (EDE [22]) compared to 44.0% of those with shorter disease duration. The rest of the patients remained unchanged, no patient deteriorated.

Building on this previous literature, the aim of the current study was to evaluate the outcome of inpatient treatment for anorexia nervosa on an individual basis using the criteria of clinical significance. We used a relativly wide range of outcome measures (body-mass-index (BMI), eating disorder symptoms, general psychopathology and depression). Furthermore, we intended to explore predictors of clinically significant changes in eating disorder psychopathology.

## Methods

### Participants

We investigated a sample of female inpatients who were hospitalised at the Schoen Klinik Roseneck in Prien, Germany between January 2009 and January 2014. Inclusion criteria were a diagnosis of anorexia nervosa according to ICD-10 (F50.00, F50.01) including intense fear of gaining weight, a distorted body image and a BMI less than or equal to 17.5. Patients were diagnosed by experienced clinicians from the highly specialized eating disorder unit (all with a minimum master’s degree in medicine or psychology) during a standard intake interview. Furthermore, a minimum age of 18 years was mandatory. Exclusion criteria were drug/alcohol/medication abuse, acute suicidal tendencies, psychotic symptoms or a severe life-threatening somatic disorder.

Patients were ‘regularly’ discharged if they achieved the target weight (BMI > 18 in accordance with eating disorder guidelines [9]). Patients who did not achieve the target weight (mostly those with a very low BMI at admission) were ‘regularly’ discharged if follow-up treatment was assured (e.g. a re-admission was planned). Patients were ‘prematurely’ discharged if they had insufficient therapy motivation or for disciplinary reasons.

All participants gave written and signed informed consent. The study was approved by the responsible Medical Ethics Committee of the University of Munich.

### Inpatient treatment program

All patients received a multimodal inpatient treatment program based on cognitive-behavioural therapy with group and individual psychotherapy. The manualised eating disorder-specific group therapy consisted of nine therapy sessions, each lasting 100 minutes. The main elements were psycho-education, behavioural and functional analysis, acceptance of one’s own body, dealing with emotions and needs, and relapse prevention. The general group psychotherapy took place up to three times a week and each session lasted 90 to 100 minutes. Patients received individual therapy once or twice a week for one hour. Additional therapy elements included social skills training, art therapy, attending classes in the teaching kitchen and exercise therapy. Patients were required to gain 700 g of body weight per week. Co-therapists weighed the patients twice a week in the morning and weight gain was visualised on charts. If patients with anorexia nervosa failed to gain weight, further steps were taken: increase of food intake and monitoring during meal times, administration of high caloric fluids or feeding through a nasal tube.

### Measures

A standard set of self-rating questionnaires were used to assess eating disorder symptomatology, general psychopathology and depression at admission and discharge.

The **Eating Disorder Inventory-2 (EDI-2)** [23,24] was used for the multidimensional assessment of the specific psychopathology of patients with eating disorders. It consists of 11 scales with 91 items that can be answered on a six-point scale from 1 (never) to 6 (always). Cronbach’s Alpha for the EDI-2 Global score for this sample was .96 at admission. Cronbach’s Alphas for the subscales are given in online supplemental materials (see Additional file 1).

The **Brief Symptom Inventory (BSI)** [25,26] assesses current general psychological distress of patients throughout the last week on the basis of 53 items belonging to nine subscales. Answers are given on a five-point scale, ranging from 0 (not at all) to 4 (extremely). Three global parameters can be calculated. In the present study, the global severity index (GSI) was used. Cronbach’s Alpha for the GSI for this sample was .96.

The **Beck Depression Inventory-2 (BDI-2)** [27,28] is a self-rating instrument to assess the severity of depressive symptoms. Patients can rate each item on a four-point scale from 0 to 3 in terms of its occurrence and its intensity during the last seven days. The cut-off for clinically relevant depressive symptoms is 20. Cronbach’s Alpha for the BDI-2 was .92.

Higher scores in these three instruments indicate greater psychopathology.

The treating therapist rated the patient’s **motivation for changing their eating disorder behaviours** at admission on a scale from 0 (not at all motivated) to 4 (highly motivated).

Additionally, sociodemographic and clinical characteristics (e.g. BMI ad admission and at discharge, duration of inpatient treatment, duration of the eating disorder, comorbidity) were available from each patient’s clinical records.

### Statistical analysis

In order to examine the effectiveness of inpatient treatment for anorexia nervosa, results were analysed in three different ways:

- Statistical group comparisons (repeated measures analyses of variance (rANOVAs) with treatment duration as covariate).
- Calculation of effect sizes.
- Assessment of treatment outcome on the basis of individual changes according to the criteria of clinical significance [14].

Effect sizes were calculated using the formula (M_pre_-M_post_)/SD_pre_. Interpretation of the effect size was corrected by the effects of an untreated control group, i.e. ES = 0.10, as proposed for single group pre-post study designs by Grawe et al. [29]. Therefore, an ES > 0.30 is considered a small effect, an ES > 0.60 a medium effect and an ES > 0.90 a large effect.

### The concept of clinical significance

The concept of clinical significance by Jacobson and colleagues [14,20] is a construct used to evaluate clinically meaningful changes resulting from therapy. It consists of a two-part criterion: To qualify as clinically significantly improved after treatment, a patient has to i) show a reliable change, i.e. statistically significant improvement and ii) cross the cut-off point for a clinically significant change.

#### The criterion of statistical significance/reliable change

To classify a patient as having shown a statistically significant change (i.e. that the observed change is not based on errors of measurement or chance), an individual minimal pre-post change (from admission to discharge) is necessary.

Therefore, the reliable-change-index (RCI) has to be exceeded, and this is calculated as follows: $RCI = \frac{{\mathrm{X}}_{\mathrm{pre}}-{\mathrm{X}}_{\mathrm{post}}}{{\mathrm{S}}_{\mathrm{diff}}}>1.96$ with ${\mathrm{S}}_{\mathrm{diff}} = \sqrt{2{\left(\mathrm{SE}\right)}^2}$ and $\mathrm{SE}={\mathrm{s}}_1\sqrt{\left(1-{\mathrm{r}}_{\mathrm{xx}}\right)}$.

Here X_pre_ represents the pre-test score and X_post_ the post-test score of a patient. Additionally, the standard error of measurement (SE), the standard deviation of the patient group at pretest (s_1_) and a measure for the reliability of the measurement (r_xx_ = Cronbach’s α) are included in this formula. S_diff_ stands for the standard error of difference between the two test scores.

#### The criterion of clinical significance

A reliable change is considered to be a precondition for a clinically relevant change. As a second step, a cut-off is defined in order to assess whether a patient should be assigned to the healthy or clinical group after treatment.

For the present study, cut-off point C according to the classification by Jacobson and Truax [14] was used. This cut-off is a weighted midpoint between the means of a functional and a dysfunctional population [30]: $C = \frac{S{D}_0*{M}_1+S{D}_1*{M}_0}{S{D}_0+S{D}_1}$. M_0_ and SD_0_ represent the mean and standard deviation of the normative sample, M_1_ and SD_1_ the mean and standard deviation of the patient group at pre-test. Norms for the healthy samples were taken from the applicable manuals [24,26,28]. Reaching the cut-off means that, following treatment, a patient is closer to the mean of a functional population than to the mean of inpatients with anorexia nervosa at admission.

According to the cut-off, patients can be classified into five treatment outcome groups:

1. **Normative:** patients with normative scores both at admission and discharge.
2. **Deteriorated:** statistically significant worsening (RCI ≤ −1.96) of patients; clinical significance is not of interest as the result is clearly unwanted.
3. **Unchanged:** patients with scores above norm at admission and statistically non-significant individual changes at discharge.
4. **Reliably improved:** statistically significant improvement (RCI ≥ 1.96) of patients between pre- and post-measurement.
5. **Clinically significantly improved:** patients with statistically significant improvement and symptoms within the normal range of a functional sample at the end of therapy.

In this study, only patients from groups 2 through 5 were considered in the analyses of each separate scale. We did not include patients with normative scores since improvement was not an issue.

To investigate differences between the different treatment outcome groups, ANOVAs or Chi-Square-Tests with post-hoc tests were calculated.

A stepwise binary logistic regression was calculated using the backward likelihood ratio method to identify predictors of clinically significant changes in the EDI-2 Global score. We divided patients into two groups: those who showed clinically significant change and those who did not (i.e. we grouped together patients who showed reliable change, patients who were unchanged, and patients who had deteriorated). We first tested for differences between these two groups in terms of sociodemographic variables, clinical characteristics and baseline subscales. In a second step, we included these variables as predictors in the regression analyses, if they showed significant differences between the patient groups and if they showed no multi-collinearity.

All statistical analyses were performed with SPSS version 22.0. The Bonferroni correction was used for multiple-comparison correction. Based on running 15 F-tests alpha was set at .003. To reflect actual clinical practice, patients who were discharged regularly, as well as those who were discharged prematurely, were included in analyses, with no replacement of missing values.

## Results

### Study sample

**Figure 1:**
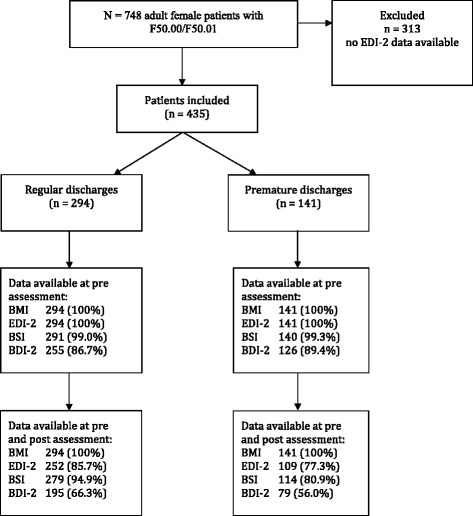
**Flow chart of patient sample.** Notes: BMI = Body-mass-index, EDI-2 = Eating disorder inventory-2, BSI = Brief symptom inventory, BDI-2 = Beck depression inventory-2.

As shown in the participant flow chart (Figure 1) between January 2009 and January 2014 748 adult female patients with anorexia nervosa (F50.00/F50.01) were treated as inpatients in the Schoen Klinik Roseneck. Due to administrative regulations, EDI-2 data were not available from n = 313 patients. There were no significant differences between those for whom EDI-2 data were available and those for whom data were unavailable in terms of BMI at admission, age, treatment duration, duration of illness, previous inpatient and outpatient treatments. Furthermore, there were no differences in BMI change during inpatient treatment between these two groups. A total of 435 inpatients with anorexia nervosa were included in the analyses of whom n = 294 regularly completed treatment and n = 141 were premature discharges (see Table 1 for more detail). Patients had a mean age of M = 26.36 (SD = 9.02). BMI at admission was 14.56 kg/m^2^ (SD = 1.74). Individual length of admission varied considerably (6–260 days) and mean duration of inpatient treatment was 91.79 days (SD = 44.26). The sociodemographic and clinical characteristics of the sample are presented in Table 1. For participants who were discharged prematurely, there were no differences in sociodemographic and clinical variables between those with available EDI-2 discharge data and those without. However, differences emerged for the patients who were discharged regularly: those with no discharge data had a lower BMI at admission, more depressive symptoms, more general psychopathology, and more previous inpatient treatments than those with discharge data.

**Table 1:** **Sociodemographic and clinical characteristics of anorexia nervosa inpatients (N = 435) at admission**

**Variables**	**M (SD)**	**Range**
Age (years)	26.36 (9.02)	18 - 70
Inpatient treatment duration (days)	91.79 (44.26)	6 - 260
Body-Mass-Index (kg/m^2^)	14.56 (1.74)	9.69 - 17.48
Duration of the eating disorder (years)	8.84 (7.81)	1 - 50
Number of previous inpatient treatment	1.86 (2.45)	0 - 15
Number of previous outpatient treatment	1.73 (1.57)	0 - 9
**Variables**	**n (%)**	
Type of discharge		
Regular	292 (67.1)	
Premature	139 (32.0)	
Prematurely discontinued by the patient	32 (7.4)	
Prematurely discharged by mutual agreement	94 (21.6)	
Insufficent insurance coverage	1 (0.2)	
Transfer	12 (2.8)	
Subtype of anorexia nervosa		
F 50.00 (Restricting Type)	316 (72.6)	
F 50.01 (Binge Eating/Purging Type)	111 (25.5)	
Comorbidity^1^		
Moderate depressive episode (F32.1)	117 (26.9)	
Severe depressive disorder^2^ (F32.2)	28 (6.4)	
Recurrent moderate depressive disorder (F33.1)	89 (20.5)	
Recurrent severe depressive disorder^2^ (F33.2)	42 (9.7)	
Social phobia (F40.1)	39 (9.0)	
Predominantly compulsive acts (F42.1)	24 (5.5)	
Posttraumatic stress disorder (F43.1)	32 (7.4)	
Emotionally unstable personality disorder (F60.3)	34 (7.8)	

Notes: ^1^Only diagnoses pertaining to more than 5% of included patients are indicated, ^2^without psychotic symptoms.

### Body weight and eating disorder symptomatology

BMI rose on average from 14.56 kg/m^2^ (SD = 1.74) to 17.18 kg/m^2^ (SD = 1.86) (ES = 1.51) during inpatient treatment. Treatment duration significantly contributed to pre-post change in body weight. The total mean weight increase was 7.33 kg (SD = 4.31). The mean weight increase per week was 0.84 kg (SD = 0.56). 52.2% of the patients remained in the anorectic BMI range (≤ 17.5) at discharge. Of 435 patients 108 (24.8%) reached a BMI ≥ 18.5 at discharge. For patients who were discharged regularly, percentages were 38.3% (BMI ≤ 17.5) and 33.1% (BMI ≥ 18.5).

As shown in Table 2, all scales of the EDI-2 indicated statistically significant changes after treatment. The largest effect sizes were found for *Drive for Thinness*, the EDI-2 *Global score* and *Interoceptive Awareness*, whereas the lowest were achieved for *Body Dissatisfaction, Interpersonal Distrust* and *Perfectionism*. Treatment duration only had a significant effect on pre-post change in *Interoceptive Awareness.*

**Table 2:** **Pre-post results regarding BMI, EDI-2, BSI and BDI-2**

	**Pretreatment**	**Posttreatment**	**rANOVAs**				**ES**
	**M (SD)**	**M (SD)**					
			**Pre-post change**		**Interaction between treatment duration and pre-post change**		
			**F (df)**	**p**	**F (df)**	**p**	
**BMI**	14.56 (1.74)	17.18 (1.86)	24.77 (1)	<.001	256.04 (1)	<.001	1.51
**EDI-2**							
Drive for Thinness	29.38 (8.80)	21.69 (8.69)	47.96 (1)	<.001	3.15 (1)	.077	0.87
Bulimia	16.95 (9.38)	10.62 (5.03)	25.50 (1)	<.001	1.97 (1)	.162	0.67
Body dissatisfaction	38.24 (9.39)	33.85 (10.49)	24.53 (1)	<.001	0.44 (1)	.510	0.47
Ineffectiveness	36.93 (10.38)	30.10 (10.60)	34.03 (1)	<.001	1.57 (1)	.211	0.66
Perfectionism	22.90 (6.20)	20.97 (5.98)	8.09 (1)	.005	0.48 (1)	.487	0.31
Interpersonal distrust	23.77 (6.69)	21.11 (6.65)	9.18 (1)	.003	2.89 (1)	.090	0.40
Interoceptive awareness	35.91 (9.52)	28.90 (9.42)	24.33 (1)	<.001	5.50 (1)	.020	0.74
Maturity fears	27.72 (7.90)	24.03 (7.43)	9.72 (1)	.002	3.33 (1)	.069	0.47
Asceticism	25.97 (7.49)	21.84 (7.26)	14.75 (1)	<.001	2.33 (1)	.128	0.55
Impulse regulation	28.36 (7.62)	24.29 (7.89)	19.62 (1)	<.001	0.63 (1)	.428	0.53
Social insecurity	28.27 (6.63)	24.84 (7.10)	19.37 (1)	<.001	0.85 (1)	.356	0.52
EDI-2 Global score	313.69 (62.85)	262.00 (66.42)	42.95 (1)	<.001	3.79 (1)	.052	0.82
**BSI GSI**	1.33 (0.70)	0.76 (0.57)	32.19 (1)	<.001	11.17 (1)	.001	0.82
**BDI-2**	28.53 (11.60)	13.97 (11.24)	52.49 (1)	<.001	3.06 (1)	.082	1.26

Notes: BMI = Body-mass-index, EDI-2 = Eating disorder inventory-2, BSI = Brief symptom inventory, GSI = Global severity index, BDI-2 = Beck depression inventory-2, ES = effect size, treatment duration as covariate.

Additional file 1 shows mean and standard deviations of community norms, the patients’ pre-treatment scores and the reliabilities used to calculate cut-offs for a clinically significant change. Furthermore, numbers of patients in the normative range for each scale are given in Additional file 1.

**Figure 2:**
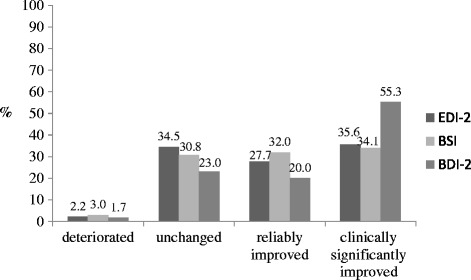
**Treatment outcome on the Global scores using the criteria of clinical significance.** Notes: EDI-2 = Eating disorder inventory-2, BSI = Brief symptom inventory, BDI-2 = Beck depression inventory-2.

Results show that 27.7% of the patients improved reliably on the *Global score* of the EDI-2. 35.6% fulfilled the criteria for a clinically significant change. In contrast, 34.5% remained unchanged and 2.2% showed worsened symptoms (see Figure 2). Additional file 2 presents the classification of treatment outcome based on the criteria of Jacobson and Truax [14] for all subscales of the EDI-2.

### General psychopathology

Change on the *GSI* of the BSI was statistically significant. Treatment duration had a significant effect on pre-post change in regard to *GSI*. The effect size was medium (ES = 0.82) (see Table 2). 32.0% of the patients showed reliable changes during inpatient treatment according to the *GSI*, 34.1% were clinically significantly improved, 30.8% unchanged and 3.0% worse (see Figure 2).

### Depression

Depressive symptoms significantly improved after treatment with a large effect size of ES = 1.26 (see Table 2). Regarding the BDI-2, the distribution of treatment outcome groups was as follows: 20.0% of patients were reliably improved, 55.3% were clinically significantly improved and 23.0% remained unchanged. 1.7% of the patients deteriorated (see Figure 2).

### Differences between deteriorated/unchanged, reliably improved and clinically significantly improved patients in regard to baseline variables

Since the number of deteriorated patients was very low, these were combined with the unchanged patients. Table 3 shows that clinically significantly improved patients had fewer symptoms of both general and eating disorder psychopathology at admission, and a lower rate of comorbid recurrent severe depression and of posttraumatic stress disorder. They were more motivated for treatment and had a higher rate of moderate depression. Deteriorated/unchanged patients showed a higher rate of comorbid recurrent severe depression and posttraumatic stress disorder.

**Table 3:** **Differences between outcome groups on the EDI-2 global score**

	**Deteriorated/unchanged (G1)**	**Reliably improved (G2)**	**Clinically significantly improved (G3)**		
	**M (SD)**	**M (SD)**	**M (SD)**	**ANOVA**	**Post-hoc**
Age (years)	25.35 (8.87)	25.94 (8.40)	26.19 (7.79)	F (2, 168) = 0.16, p = .853	G1 = G2 = G3
Inpatient treatment duration (days)	88.56 (42.01)	103.41 (37.82)	86.37 (34.78)	F (2, 168) = 3.03, p = .051	G3 = G1 = G2
Body-Mass-Index (kg/m^2^) at admission	14.65 (1.54)	14.75 (1.80)	14.95 (2.02)	F (2, 168) = 0.44, p = .645	G1 = G2 = G3
Duration of the eating disorder (years)	8.27 (6.77)	8.65 (8.09)	8.50 (6.78)	F (2, 168) = 0.04, p = .958	G1 = G3 = G2
Number of previous inpatient treatment	1.96 (1.64)	1.75 (1.47)	1.73 (1.33)	F (2, 168) = 0.43, p = .649	G3 = G2 = G1
Number of previous outpatient treatment	2.32 (2.88)	2.10 (2.34)	1.29 (1.54)	F (2, 168) = 2.96, p = .055	G3 = G2 = G3
Motivation	2.09 (1.11)	2.43 (1.17)	2.54 (1.07)	F (2, 213) = 3.45, p = .034	G2 = G1 < G3
BDI-2 at baseline	30.34 (10.83)	37.51 (9.11)	27.21 (10.07)	F (2, 168) = 14.13, p < .001	G3 = G1 < G2
BSI at baseline	1.56 (0.66)	1.87 (0.51)	1.24 (0.59)	F (2, 168) = 14.01, p < .001	G3 < G1 < G2
EDI-2 Global score at baseline	333.59 (46.52)	373.10 (32.55)	305.77 (33.60)	F (2, 168) = 38.78, p < .001	G3 < G1 < G2
	**n (%)**	**n (%)**	**n (%)**	**χ** ^**2**^ **-Test**	**Post-hoc**
Comorbidity					
Moderate depressive episode (F32.1)	33 (32.4)	18 (23.4)	41 (41.4)	χ^2^ (2, 278) = 6.40, p = .041	G2 < G3
					G1 = G3
					G2 = G1
Recurrent severe depressive disorder^1^ (F33.2)	12 (11.8)	15 (19.5)	4 (4.0)	χ^2^ (2, 278) = 10.48, p = .005	G3 < G2 = G1
					G3 = G2
Posttraumatic stress disorder (F43.1)	13 (12.8)	6 (7.8)	2 (2.0)	χ^2^ (2, 278) = 8.28, p = .016	G3 < G1
					G2 = G1
				**Kruskal-Wallis-Test**	**Post-hoc**
Education					
Still in school	4 (3.9)	3 (3.9)	9 (9.1)	H(2) = 7.34, p = .026	G3 = G1
					G1 = G2
No graduation	1 (1.0)	-	-		
Secondary general school certificate	7 (6.9)	4 (5.2)	7 (7.1)		G3 < G2
Intermediate school certificate	25 (24.5)	13 (16.9)	30 (30.3)		
Higher education entrance qualification	64 (62.7)	56 (72.7)	53 (53.5)		

Notes: G1 = Group 1, G2 = Group 2, G3 = Group 3, ^1^without psychotic symptoms. Only comorbidities with significant differences are presented.

### Predictors for a clinically significant change in the *Global score* of the EDI-2

A higher motivation for change as well as more depressive symptoms turned out to be significant predictors for a clinically significant change whereas body dissatisfaction, impulse regulation, social insecurity and education were negative predictors (see Table 4). These factors explained 35.0% of the variance in scores (Nagelkerke’s R^2^).

**Table 4:** **Predictors of a clinically significant change of EDI-2 global score**

	**ß**	**S.E.**	**Wald**	**p**	**OR (95% CI)**
EDI-2 Body dissatisfaction	-.070	.02	9.38	.002	0.93 (0.89-0.98)
EDI-2 Impulse regulation	-.066	.03	4.26	.039	0.94 (0.88-1.00)
EDI-2 Social insecurity	-.088	.04	5.23	.022	0.92 (0.85-0.99)
Moderate depressive episode (F32.1)	1.049	.39	7.21	.007	2.85 (1.33-6.13)
Education	-.475	.20	5.69	.017	0.62 (0.42-0.92)
Motivation for change	.401	.18	5.19	.023	1.49 (1.06-2.11)

Note: EDI-2 = Eating disorder inventory-2.

## Discussion

The aim of this study was to evaluate the effectiveness of inpatient treatment for anorexia nervosa according to the criteria of clinical significance of Jacobson and colleagues [14,20]. This construct allowed detailed analyses of treatment outcome on an individual basis. The results showed that – depending on the outcome measure – one-third to more than one half of the patients showed clinically significant changes. Furthermore, the rate of patients with reliable changes was more than 30%.

Lambert and Ogles [31] assume that 25-30% of patients generally do not change during psychotherapy and that about 5-10% even worsen during treatment. Our non-response rates are somewhat higher than the ones reported by these authors, while deterioration rates are somewhat lower.

Our rate of clinically significant improvement following inpatient treatment for anorexia nervosa is comparable to the findings of Calugi and colleagues [21], whereas the non-response rate found in our sample was only half of that in the comparison study. In contrast to our study, which used the self-rated EDI-2 *Global score* to evaluate treatment outcome, Calugi and colleagues [21] used the EDE semi-structured interview. Therefore, our self-rating instrument might have overestimated effects compared to the expert rating.

The high effect size of ES = 1.51 for the BMI is larger than a meta-analytically calculated effect size of inpatient treatment for anorexia nervosa with ES = 1.19 (CI: 1.07 - 1.30) [32]. However, despite the considerable weight gain, 52.2% of patients remained in the anorectic BMI range at discharge. This result is comparable with Goddard and colleagues [19] who also found that more than half of their patients did not reach a BMI > 17.5 during inpatient treatment. One explanation is the severity of anorexia nervosa in inpatient samples. In our sample individuals suffered from long-term anorexia nervosa (mean illness duration of almost 10 years) and had a rather low BMI at admission.

For depression, as measured by the BDI-2, the large effect size is consistent with the finding by Bowers et al. [10]. The medium effect sizes found for the *Global scores* of EDI-2 and the general psychopathology in this study are in line with effects shown in previous studies examining the effectiveness of inpatient treatment for anorexia nervosa [12,13,33,34].

Treatment duration had a significant effect on BMI change as well as on changes in *Interoceptive Awareness* and general psychopathology *(GSI).* Other subscales were not significantly influenced by treatment duration. This is in line with results from Morris et al. [11], who found that length of inpatient stay was significantly correlated with BMI change but not with change in EDE questionnaire. Collin et al. [12] also reported that length of current admission was a predictor of BMI change, but not of EDE change or change in the SCL-90 (Symptom Checklist-90) global severity index [35].

When comparing deteriorated/unchanged, reliably improved and clinically significantly improved patients, differences in regard to numerous baseline variables were found. Clinically significantly improved patients were less severely ill at admission, i.e. they showed less symptoms in terms of general and eating disorder psychopathology at admission and had a lower rate of comorbid disorders (recurrent severe depression, posttraumatic stress disorder). One plausible explanation as to why these patients had the highest rate of clinically significant changes is that lower scores at baseline make it easier to reach the cut-off for a clinically significant change. Clinically significantly improved patients were also more motivated, which may also explain why they showed the most pronounced treatment effects. Comorbid depression was also observed in patients with clinically significant changes. A positive protective effect of depression was also found by Zeeck et al. [36], who reported that inpatients with anorexia nervosa and comorbid depression stayed longer in psychotherapy than those without comorbid depression. These patients therefore have a higher chance for clinically significant changes. Deteriorated/unchanged patients showed a higher rate of severe comorbid conditions (recurrent severe depression, posttraumatic stress disorder).

The regression analysis emphasized the relevance of high internal motivation for change as a predictive factor for a better treatment outcome at the time of discharge. Previous studies have also found that a higher baseline motivation is an important predictor for change in anorexia nervosa inpatients [37,38]. These results suggest that motivational strategies should be a key starting point for improving the effectiveness of inpatient treatment for anorexia nervosa. One possible future approach is to increase the patients’ readiness for change, for example using Motivational Interviewing techniques [39] which also showed promise for the treatment of patients with eating disorders [40]. Furthermore, body dissatisfaction was identified as a negative predictor of a clinically significant change. This is in line with results from other studies which have found that body dissatisfaction is a negative predictor of weight increase [41,42]. In our study, impulse regulation was a negative predictor of short-term outcome. Fichter et al. [43] also found that impulse regulation was a negative predictor at 12 year follow-up. While Karllson et al. [41] found that social insecurity was a positive predictor for weight gain, we found that it was a negative predictor for clinically significant change. It may be that social insecurity leads to reduced therapeutic alliance and therefore a less favourable treatment outcome. A comorbid moderate depressive episode was found to be a positive predictor of clinically significant change. This contrasts with previous studies which have shown that depressive symptoms are a negative predictor of treatment outcome [43]. Furthermore, Calugi et al. [44] showed that there were no differences in outcome between eating disorder patients with or without a comorbid major depressive disorder. Our results should be interpreted carefully since comorbidity was not assessed by a structured clinical interview such as the Structured Clinical Interview for DSM Disorders [45], but rather based on expert opinion. When comparing values of depressive symptoms among the unchanged, the reliably improved and clinically significantly improved patients in our study, results showed that clinically significantly improved patients had the lowest depressive symptoms. Finally, a higher education was identified as negative predictor for treatment outcome. This contrasts with findings from Huas et al. [46], who found that low educational status was a predictor of dropout from inpatient treatment for anorexia nervosa, which is linked in general to poorer outcomes.

### Strengths and limitations

To the best of our knowledge, this study includes the largest sample size of anorexia nervosa patients treated as inpatients. Furthermore, strength of the study is that it also evaluates individual treatment outcomes. This enables a more realistic view of treatment outcomes than comparisons of means and effect sizes do [47]. A further advantage of the study is the naturalistic sample. In comparison to data from randomised controlled trials, data from naturalistic settings may yield more clinically useful findings [48] because they represent the heterogeneous nature of the cases, settings, referral routes, and therapists that characterise everyday clinical practice [49]. These factors enable more valid conclusions to be drawn about treatment improvement.

The strength of the clinical significance concept lies in its applicability to individual cases. Furthermore, evaluation of each individual patient with regard to normative functioning is of great value for clinical practice [30].

There are several limitations. First, the data do not represent all patients with anorexia nervosa admitted in the defined period but rather those for whom baseline and discharge questionnaires were available (about 50%). There were no differences between those with and without missing baseline data in terms of sociodemographic and clinical variables. Furthermore, there were no differences in BMI change during inpatient treatment. However, there were differences between those with and without discharge questionnaires. Since patients who did not complete discharge assessment were more severely ill, results may have overestimated treatment effects. On the other hand, we included outcome data from patients who were prematurely discharged so that our results may after all give a conservative evaluation of treatment outcome. Analyses of only those patients who were discharged regularly might have revealed more positive outcomes. Second, all data except those for the BMI, were based on self-ratings, and no structured clinical interview was conducted. Diagnoses were finally fixed at the end of treatment, giving the expert rater a broad vision of the patient’s behaviour. Third, no follow-up data could be collected to provide data on the long-term outcome of inpatient treatment for anorexia nervosa. Fourth, although one item rated therapy motivation as a predictor of treatment outcome, this has neither been validated against other measures that assess motivation, nor has its reliability been established. One study assessing readiness to change (University of Rhode Island Change Assessment, [50]) in inpatients with anorexia nervosa found that BMI change was not predicted by the stage of change of the patients [51]. A further limitation of the patient’s motivation being rated by the therapist is that the rating data may reflect the motivation of the therapist to some extent, and may influence the motivation of the therapist and consequently treatment quality. Fifth, the method of clinical significance has weaknesses. A statistical limitation of this approach is regression to the mean. Patients with higher scores at admission are those most likely to make huge improvements [52]. Also cut-off point C as a cut-off point between normal and dysfunctional distribution can vary considerably among studies as this criterion depends on the symptom severity in the study sample at baseline. Furthermore, the use of the two criteria to evaluate clinically significant change represents a more stringent measure than most of the previously used criteria. Moreover, one needs to take into account that treatment is, in most cases, not completed when inpatient care concludes. In most cases, inpatient psychotherapy represents a specific phase of a long-term treatment process. Consequently, additional outpatient therapy is required to ensure further improvements of symptoms and a long-term stabilization of achieved effects. There is one caveat, however: patients with anorexia nervosa are often chronically ill and becoming completely symptom-free might be unrealistic, even in the long run.

The paradigm of ‘clinically relevant treatment effects’ is worthy of some discussion in general. Self-ratings that focus on the assessment of symptoms are certainly important in this context, but it is still only one aspect that must be complemented by other perspectives (e.g. expert ratings). Other factors may equally well indicate clinically relevant treatment effects (e.g. acceptance of disease, improved quality of life, increased knowledge and capability to deal with one’s own illness, provision of a basis for improvement of symptoms in the ensuing outpatient therapy).

Although inpatient treatment for anorexia nervosa is effective, a certain percentage of patients do not benefit from it – at least in certain symptom areas. There are several reasons for the absence of significant or clinically relevant treatment effects. For example, a lack of motivation may reduce response to treatment. Additionally, a lack in quality of the therapy (e.g. a poor therapeutic relationship, overestimation of the patient’s capabilities) may contribute to non-response. Moreover, adverse external events that occur during inpatient treatment such as relationship breakups, job loss, emerging conflicts, may result in non-response or even a deterioration of symptoms in some patients.

Finally, there is the question of how the concept of clinical significance can be integrated in other definitions of treatment outcome. In outcome research there are many different terms used to describe treatment outcome, e.g. response, (partial) remission, recovery and relapse that are often differently defined [53]. In eating disorders research there are also a variety of definitions of outcome success that are used [54–57]. Couturier et al. [54] reviewed various conceptualizations of remission in adolescent anorexia nervosa and reached the conclusion that remission is best defined as the attainment of a certain weight and/or the attainment of normal EDE scores (within 1 or 2 SD of the normal range, depending on the distribution). Bardone-Cone et al. [57] defined recovery as no longer meeting diagnostic criteria for anorexia nervosa, showing a BMI ≥ 18.5, no binging or purging, and EDE scores within 1 SD of community norm for each subscale. All these definitions compare individuals’ outcomes with a norm at a predefined assessment time. However, they do not consider changes resulting from treatment. As Frank et al. [53] stated, remission and recovery are an assessment of outcome at a single point and are not entirely dependent on treatment. However, response is a change due to treatment that requires baseline and post-treatment scores. In this sense, the first criterion of the concept of clinical significance (reliable change) assesses response to treatment. A clinically significant change requires both response to treatment as well as achieving a functional range at endpoint assessment. A clinical significant change may therefore be seen as remission, but considers response to treatment, too. Therefore, the criteria of clinical significance may be a more appropriate definition for evaluating treatment outcome.

### Future research

In order to optimize treatment, future studies should explore the complex interplay of factors that may prevent patients with anorexia nervosa from non-responding to inpatient treatment. Then, treatment strategies for those should be developed. For those who improved during inpatient treatment, the challenge remains in maintaining therapy success following discharge. More attention should be paid to relapse prevention strategies, of which technology-based relapse prevention may be particularly useful [58,59]. Additional research should also address clarifying the impact of depressive symptoms or of depressive comorbidity on outcome for anorexia nervosa patients. Furthermore, future research should also investigate if patients’ therapy motivation or stage of change as assessed by validated disorder-specific measures [60–62] are a predictor for a clinically significant change.

## Conclusions

Inpatient treatment of anorexia nervosa is highly effective in restoring weight. A good proportion of patients show reliable changes in terms of eating disorder psychopathology, general psychopathology and depression. However, a significant proportion of non-responders remain. Supplementing group statistics with analyses of outcome on an individual level is suitable for obtaining a more precise picture of treatment outcome and is of immense practical relevance.

## Additional files

Additional file 1:
**Pre-treatment scores of patients, community norms, Cronbach’s Alpha and criterion C for each outcome measure.**
Additional file 2:
**Classification of treatment outcome (EDI-2 subscales) based on the criteria of Jacobson and Truax** [14].

## Abbreviations

BMI: Body-mass-index
EDE: Eating disorder examination
ICD-10: International statistical classification of diseases and related health problems
EDI-2: Eating disorder inventory-2
BSI: Brief symptom inventory
GSI: Global severity index
BDI-2: Beck depression inventory-2
ES: Effect size
Mpre: Mean pre (at admission)
Mpost: Mean post (at discharge)
SDpre: Standard deviation pre (at admission)
RCI: Reliable change index
SPSS: Statistical package for social sciences
G1: Group 1 (deteriorated/unchanged patients)
G2: Group 2 (reliably improved patients)
G3: Group 3 (clinically significantly improved patients)
