# Supplementary material for: Specialized inpatient treatment of adult anorexia nervosa: effectiveness and clinical significance of changes
Source: BMC Psychiatry. 2014 Sep 6;14:258. doi: 10.1186/s12888-014-0258-z (PMC4172844; doi:10.1186/s12888-014-0258-z)
Supplement: Additional file 1: — Pre-treatment scores of patients, community norms, Cronbach’s Alpha and criterion C for each outcome measure. [file 12888_2014_258_MOESM1_ESM.pdf]

Additional file 1: Pre-treatment scores of patients, community norms, Cronbach's Alpha and criterion C for each outcome measure

	N Patients	M Patients	SD Patients	M Norm	SD Norm	Cronbach's α Patients	Criterion C	% Normative patients
**EDI-2**								
Drive for Thinness	434	29.60	8.65	17.3	6.8	.89	22.71	21.8
Bulimia	434	16.73	9.26	10.6	3.4	.92	12.25	41.7
Body Dissatisfaction	429	38.21	9.40	30.2	10.3	.85	34.39	37.0
Ineffectiveness	429	37.33	10.78	23.5	5.7	.91	28.28	21.3
Perfectionsim	434	23.17	6.27	16.5	5.7	.77	19.68	25.1
Interpersonal Mistrust	432	24.01	6.89	18.4	4.4	.84	20.59	29.6
Interoceptive Awareness	434	36.25	9.63	22	5.7	.84	27.30	16.9
Maturity Fears	431	27.78	8.04	20.8	4.7	.83	23.37	26.9
Asceticism	429	26.23	7.51	16.5	4	.78	19.88	17.3
Impulse regulation	427	28.75	8.10	23.6	6.3	.77	25.85	36.4
Social Insecurity	433	28.52	6.86	22	4.7	.78	24.65	26.4
EDI-2 Global score	433	315.96	63.86	223.2	38.1	.96	257.86	20.6
**BSI GSI**	431	1.37	0.72	0.35	0.23	.96	0.60	15.7
**BDI-2**	381	28.37	12.22	7.4	7.3	.92	15.24	13.9

Notes: EDI-2=Eating disorder inventory-2, BSI=Brief symptom inventory, GSI=Global severity index, BDI-2=Beck depression inventory-2, norms for the healthy samples were taken from the applicable norms [24, 26, 28]
