# Supplementary material for: Specialized inpatient treatment of adult anorexia nervosa: effectiveness and clinical significance of changes
Source: BMC Psychiatry. 2014 Sep 6;14:258. doi: 10.1186/s12888-014-0258-z (PMC4172844; doi:10.1186/s12888-014-0258-z)
Supplement: Additional file 2: — Classification of treatment outcome (EDI-2 subscales) based on the criteria of Jacobson and Truax [14]. [file 12888_2014_258_MOESM2_ESM.pdf]

Additional file 2: Classification of treatment outcome (EDI-2 subscales) based on the criteria of Jacobson and Truax [14]

	**Deteriorated (%)**	**Unchanged (%)**	**Reliably improved (%)**	**Clinically significantly improved (%)**
Drive for Thinness	3 (1.1)	135 (48.7)	48 (17.3)	91 (32.9)
Bulimia	1 (0.5)	87 (42.9)	38 (18.7)	77 (37.9)
Body Dissatisfaction	6 (2.8)	152 (70.7)	16 (7.4)	41 (19.1)
Ineffectiveness	4 (1.5)	156 (57.1)	36 (13.2)	77 (28.2)
Perfectionism	5 (2.0)	232 (90.6)	7 (2.7)	12 (4.7)
Interpersonal Distrust	5 (2.1)	193 (79.4)	16 (6.6)	29 (11.9)
Interoceptive Awareness	3 (1.0)	188 (64.6)	40 (13.7)	60 (20.6)
Maturity Fears	2 (0.8)	189 (77.5)	17 (7.0)	36 (14.8)
Asceticism	4 (1.4)	213 (73.2)	34 (11.7)	40 (13.7)
Impulse Regulation	2 (0.9)	174 (77.7)	6 (2.7)	42 (18.8)
Social Insecurity	4 (1.6)	201 (78.2)	12 (4.7)	40 (15.6)
